# Supplementary material for: Effectiveness and Safety of Ayurvedic Medicines in Type 2 Diabetes Mellitus Management: A Systematic Review and Meta-Analysis
Source: Front Pharmacol. 2022 Jun 8;13:821810. doi: 10.3389/fphar.2022.821810 (PMC9213670; doi:10.3389/fphar.2022.821810)
Supplement: Supplementary file 1 [file DataSheet1.zip › Supplementary Material/Forest and Funnel Plots/Plantago ovata/Fasting insulin.pdf]

Plantago ovata - Fasting insulin

Study	Intervention N	Intervention Mean	Intervention SD	Comparator N	Comparator Mean	Comparator SD	Mean Diff. with 95% CI	Weight (%)
Ziai 2005	21	7.40	5.04	15	9.10	3.10	-1.70 [-4.58, 1.18]	49.06
Abutair 2016	18	19.70	2.90	18	24.90	5.13	-5.20 [-7.92, -2.48]	50.94
**Overall**							-3.48 [-6.91, -0.05]	

Heterogeneity: $\tau^2 = 4.08$, $I^2 = 66.62\%$, $H^2 = 3.00$

Test of $\theta_i = \theta_j$: Q(1) = 3.00, p = 0.08

Test of $\theta = 0$: z = -1.99, p = 0.05

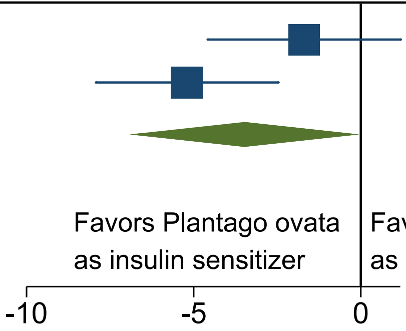

Random-effects REML model
